# Evaluation of Proton Therapy Reirradiation for Patients With Recurrent Head and Neck Squamous Cell Carcinoma

**DOI:** 10.1001/jamanetworkopen.2022.50607

**Published:** 2023-01-23

**Authors:** Anna Lee, Robbie Woods, Amgad Mahfouz, Sarin Kitpanit, Olivia Cartano, Nader Mohamed, Irini Youssef, Kathryn Marqueen, Kevin Sine, Dennis Mah, Brian Neal, Kaveh Zakeri, Jung J. Kang, Nadeem Riaz, Yao Yu, Sean M. McBride, Linda D. Chen, C. Jillian Tsai, Daphna Y. Gelblum, Robert H. Press, Loren S. Michel, Eric J. Sherman, David Pfister, Lara A. Dunn, Alan L. Ho, James Fetten, Richard J. Wong, Jay O. Boyle, Bhuvanesh Singh, Jennifer R. Cracchiolo, Ian Ganly, Marc A. Cohen, Nancy Y. Lee

**Affiliations:** 1 Department of Radiation Oncology, Memorial Sloan Kettering Cancer Center, New York, New York; 2 Department of Radiation Oncology, University of Texas MD Anderson Cancer Center, Houston, Texas; 3 Department of Surgery, Memorial Sloan Kettering Cancer Center, New York, New York; 4 ProCure Proton Therapy Center, Somerset, New Jersey; 5 New York Proton Center, New York; 6 Department of Medicine, Memorial Sloan Kettering Cancer Center, New York, New York

## Abstract

**Importance:**

Use of proton therapy reirradiation (PT-ReRT) for head and neck cancer is increasing; however, reports are heterogenous and outcomes can be difficult to interpret.

**Objective:**

To evaluate outcomes and toxic effects following PT-ReRT in a uniform and consecutive cohort of patients with head and neck squamous cell carcinoma.

**Design, Setting, and Participants:**

This retrospective cohort study included patients with recurrent primary head and neck squamous cell carcinoma who were treated with PT-ReRT from January 1, 2013, to December 31, 2020, at a single institution. Patient, clinical, and treatment characteristics were obtained, and multidisciplinary review was performed to record and grade early and late toxic effects.

**Exposures:**

Proton therapy reirradiation.

**Main Outcomes and Measures:**

Follow-up was defined from the start of PT-ReRT. The Kaplan-Meier method was used for outcomes of interest, including local control (LC), locoregional control, distant metastatic control, progression-free survival, and overall survival (OS). Cox proportional hazards regression modeling was used to assess associations of covariates with OS.

**Results:**

A total of 242 patients (median [range] age, 63 [21-96] years; 183 [75.6%] male) were included. Of these patients, 231 (95.9%) had a Karnofsky performance status score of 70 or higher, and 145 (59.9%) had at least a 10–pack-year smoking history. Median (range) follow-up was 12.0 (5.8-26.0) months for all patients and 24.5 (13.8-37.8) months for living patients. A total of 206 patients (85.1%) had recurrent disease vs second primary or residual disease. The median (range) interval between radiation courses was 22 (1-669) months. Median PT-ReRT dose was 70 cobalt gray equivalents (CGE) for the fractionated cohort and 44.4 CGE for the quad shot cohort. For the fractionated cohort, the 1-year LC was 71.8% (95% CI, 62.8%-79.0%) and the 1-year OS was 66.6% (95% CI, 58.1%-73.8%). For the quad shot cohort, the 1-year LC was 61.6% (95% CI, 46.4%-73.6%) and the 1-year OS was 28.5% (95% CI, 19.4%-38.3%). Higher Karnofsky performance status scores (hazard ratio [HR], 0.50; 95% CI, 0.25-0.99; *P* = .046) and receipt of salvage surgery prior to PT-ReRT (HR, 0.57; 95% CI, 0.39-0.84; *P* = .005) were associated with improved OS, whereas receipt of quad shot (HR, 1.97; 95% CI, 1.36-2.86; *P* < .001) was associated with worse OS. There were a total of 73 grade 3 and 6 grade 4 early toxic effects. There were 79 potential grade 3, 4 grade 4, and 5 grade 5 late toxic effects.

**Conclusions and Relevance:**

The findings of this cohort study suggest that, compared with previous reports with photon-based reirradiation, patients are living longer with aggressive PT-ReRT; however, surviving patients remain at risk of early and late complications.

## Key Points

**Question:**

What are the outcomes of patients receiving proton therapy reirradiation for head and neck squamous cell carcinoma (HNSCC)?

**Findings:**

This cohort study of 242 patients with HNSCC found 1-year local control of 71.8% and 1-year overall survival of 66.6% for those receiving fractionated reirradiation. The study found 79 potential grade 3, 4 grade 4, and 5 grade 5 late toxic effects.

**Meaning:**

These findings suggest that proton therapy reirradiation may be associated with improved survival for patients with HNSCC, but the toxic effects may be substantial.

## Teaser

This cohort study of patients with head and neck squamous cell carcinoma examines the outcomes and toxic effects associated with proton therapy reirradiation for patients with head and neck squamous cell carcinoma.

## Introduction

Head and neck cancers represent more than 500 000 new cases annually worldwide.^1^ Despite best efforts, approximately 40% of patients will develop a second cancer or locoregional recurrence.^2,3,4^ Given that locoregional recurrence is the most common cause of death in head and neck cancer,^5^ local treatment plays an important role in abating progression of disease. Unfortunately, additional treatment of a previously irradiated field can be challenging due to dosimetric limitations of organs at risk and is associated with high treatment-related toxic effects. Furthermore, patients who survive the recurrence with salvage reirradiation often experience long-term effects with high morbidity, impairing quality of life.

The use of proton therapy in this setting has been shown to be beneficial by harnessing the power of the Bragg peak, where most of the dose is deposited in the target followed by a steep dose decrease, thereby limiting dose to normal tissues outside the treatment field. Proton therapy is increasingly used as an accepted form of reirradiation to the head and neck to mitigate the complications of the normal tissues that are associated with a second course of radiation. Due to the heterogenous group of patients with head and neck cancer with varying histologic types, the outcomes of reirradiation can be difficult to interpret. Therefore, the purpose of this study was to report outcomes and toxic effects from a single institution of a uniform and consecutive cohort of patients with head and neck squamous cell carcinoma (HNSCC) who received proton therapy reirradiation (PT-ReRT), where the target has also received previous curative intent radiation.

## Methods

In this retrospective cohort study, a consecutive and uniform cohort of patients who underwent PT-ReRT for their recurrent or second primary HNSCC in a previously irradiated field at Memorial Sloan Kettering Cancer Center (MSKCC) from January 1, 2013, to December 31, 2020, formed the basis for this study. The study was approved by the MSKCC Institutional Review Board, which approved a waiver of informed consent because the study posed minimal risk to study participants. All data were deidentified. Guidelines established by the Strengthening the Reporting of Observational Studies in Epidemiology (STROBE) for cohort studies were followed.

All patients underwent proton therapy at the ProCure Proton Therapy Center in Somerset, New Jersey, an affiliate of our institution. There was no limit placed on the interval between radiation courses, and reirradiation was determined by a clinically significant overlap in the head and neck region defined by the treating physician.

Patient, clinical, and treatment characteristics were tabulated, and the median, IQR, range, numbers, and percentages were reported. Data collected included age, sex, Karnofsky performance status (KPS) score, smoking status (never, <10 pack-years, or ≥10 pack-years), original disease site (lip and oral cavity, oropharynx, skin, larynx or hypopharynx, nasal cavity or paranasal sinuses, unknown primary, nasopharynx, or salivary gland), prior radiation dose, proton reirradiation dose, proton fractionation scheme (fractionated vs quad shot), interval between radiation treatments, salvage surgery, systemic therapy, disease status at the time of PT-ReRT (locoregional, distant metastases, or both), recurrence type (recurrence, second primary, residual, or metastasis), and retreatment site (pharyngeal mucosa, neck, auricular region, skull base, orbit, cheek, or scalp). Data on race and ethnicity were not collected because they are outside the scope of this study.

All patients underwent computed tomography simulation with positron emission tomography and magnetic resonance imaging fusion when available to delineate target volumes. Preoperative imaging was fused if salvage surgery occurred. The clinical target volume, which includes the gross tumor volume or the postoperative bed, was specifically defined for each patient. Elective subclinical areas were not routinely treated given that these patients received prior irradiation to these regions. Concurrent systemic therapy was administered at the discretion of the treating physicians. Proton therapy was delivered with either 3-dimensional proton therapy using uniform scanning or pencil beam scanning, with the latter consisting of either single-field uniform dose or multifield optimization treatment planning. A relative biological effectiveness of 1.1 was used for proton dose calculations.

All early and late toxic effects were graded according to the Common Terminology Criteria for Adverse Events, version 4.0. An early toxic effect was defined as an adverse event happening during or within 3 months following completion of PT-ReRT, and a late toxic effect was any effect beyond this period. Detailed toxicity profiles were independently collected by multidisciplinary research fellows in both radiation oncology and head and neck surgery to determine consensus attribution of sequelae possibly related to proton therapy.

Local failure was defined as recurrence in the treatment field, and regional failure was defined as recurrence outside the treatment field or in a regional lymph node. Distant failure was defined as any new metastatic disease or progression of existing metastatic disease. Local control (LC), locoregional control (LRC), distant metastatic control (DMC), progression-free survival (PFS), and overall survival (OS) were calculated from the start of PT-ReRT. Variables included were age, sex, KPS, smoking status, disease status at the time of reRT, interval between radiation courses, salvage surgery, concurrent systemic treatment with reirradiation (reRT), and reRT fractionation (quad shot vs fractionated). Additional variables evaluating covariates on LC included median dose of first- and second-course radiation therapies.

### Statistical Analysis

The Kaplan-Meier method was used to estimate time-to-event end points. Cox proportional hazards regression modeling was used to assess effects of covariates on OS. Variables with a 2-sided *P* < .10 on univariable analysis were included in the multivariable model. *P* ≤ .05 was considered statistically significant. Statistical analyses were conducted in SPSS software, version 23 (SPSS Inc). The follow-up interval was defined from the beginning of PT-ReRT to the last follow-up or death.

## Results

A consecutive cohort of 242 patients with HNSCC (median [range] age, 63 [21-96] years; 183 [75.6%] male and 59 (24.4%) female) in a previously irradiated field treated with proton therapy were included in the study. Of these patients, 231 (95.9%) had a KPS score of 70 or higher, and 145 (59.9%) had at least a 10–pack-year smoking history. The most common original disease sites were lip and oral cavity (91 [37.6%]), oropharynx (64 [26.4%]), skin (27 [11.2%]), larynx and/or hypopharynx (24 [9.9%]) and other, including nasal cavity or paranasal sinus, unknown primary site, nasopharynx, or salivary gland (36 [14.9%]).

At the time of reirradiation treatment, 212 patients (87.6%) had locoregional disease, 6 patients (2.5%) had distant metastases, and 24 (9.9%) had both. A total of 206 patients (85.1%) had recurrent disease, whereas 36 (14.8%) had either a second primary or residual disease. One hundred fifty patients (62.0%) were treated in a mucosal site. Fifty-four (22.3%) were treated in the oropharynx, 53 (21.9%) were treated in the lip and oral cavity, 21 (8.7%) were treated in the larynx and/or hypopharynx, 17 (7.0%) in the nasal cavity and/or paranasal sinuses, and 5 (2.1%) in the nasopharynx. Of the entire cohort, 51 patients (21.1%) had subsequent treatment to the neck, 20 (8.3%) received treatment to the auricular region, and 11 (4.5%) to the skull base. A total of 98 patients (40.5%) received salvage surgery before reirradiation, and 138 (57.0%) received concurrent systemic therapy.

The most common concurrent chemotherapy regimens were cisplatin (49 [20.2%]), cetuximab (17 [13.6%]), cetuximab and docetaxel (33 [7.0%]), and carboplatin and paclitaxel (16 [6.6%]). The median prior radiotherapy dose was 6996 cGy. A total of 186 patients (76.9%) initially received photon-based irradiation, whereas 22 (9.1%) received proton therapy. Median (range) follow-up for those receiving fractionated reirradiation was 18 (0-88) months for the total cohort and 25 (0-88) months for those still living. Median (range) follow-up for those receiving quad shot reirradiation was 7 (0-76) months for the total cohort and 18 (0-76) months for those still living. The median (range; IQR) interval between radiation courses was 22 (1-669; 11-69) months, and the median (IQR) proton reirradiation dose was 70 (66-70) cobalt gray equivalents (CGE) for the fractionated cohort and 44.4 (18.5-44.4) CGE in the quad shot cohort, with a median (range) of 3 (1-4) cycles received. A total of 154 patients (63.6%) received fractionated radiation, and 88 (36.4%) received the quad shot regimen of 4 treatments delivered over 2 days at 3.7 CGE per fraction twice daily at 2- to 4-week intervals.^6^ Among those who received the quad shot regimen, 78 (88.6%) had unresectable disease compared with 66 (42.9%) in the fractionated group. Details can be found in Table 1.

**Table 1.** Patient, Disease, and Treatment Characteristics

Characteristic	Finding (N = 242)
Sex, No. (%)	
Male	183 (75.6)
Female	59 (24.4)
KPS score, No. (%)^a^	
<70	10 (4.1)
≥70	231 (95.9)
Smoking or tobacco use, No. (%)	
Never	72 (29.8)
<10 Pack-years	25 (10.3)
≥10 Pack-years	145 (59.9)
Original disease site, No. (%)	
Lip and oral cavity	91 (37.6)
Oropharynx	64 (26.4)
Skin	27 (11.2)
Larynx or hypopharynx	24 (9.9)
Nasal cavity or paranasal sinuses	15 (6.2)
Unknown primary	12 (5.0)
Nasopharynx	6 (2.5)
Salivary gland	3 (1.2)
First-course irradiation modality, No. (%)	
Photon irradiation	186 (76.9)
Proton irradiation	22 (9.1)
Other or missing	34 (14.0)
Disease status at the time of reirradiation, No. (%)	
Locoregional	212 (87.6)
Distant metastases	6 (2.5)
Both	24 (9.9)
Recurrence type, No. (%)	
Recurrence	206 (85.1)
Second primary	34 (14.0)
Residual or metastasis	2 (0.8)
Subsequent treatment site, No. (%)	
Pharyngeal mucosa	150 (62.0)
Oropharynx	54 (22.3)
Lip and oral cavity	53 (21.9)
Larynx or hypopharynx	21 (8.7)
Nasal cavity or paranasal sinus	17 (7.0)
Nasopharynx	5 (2.1)
Neck	51 (21.1)
Auricular region	20 (8.3)
Skull base	11 (4.5)
Orbit	6 (2.5)
Cheek	2 (0.8)
Scalp	2 (0.8)
Salvage surgery, No. (%)	
No	144 (59.5)
Yes	98 (40.5)
Concurrent systemic treatment with reirradiation, No. (%)	
No	104 (43.0)
Yes	138 (57.0)
Reirradiation fractionation, No. (%)	
Fractionated	154 (63.6)
Quad shot	88 (36.4)
Proton therapy treatment year, No. (%)	
2013	4 (1.7)
2014	27 (11.2)
2015	28 (11.6)
2016	49 (20.2)
2017	38 (15.7)
2018	43 (17.8)
2019	44 (18.2)
2020	9 (3.7)
Age at reirradiation, median (IQR), y	63 (55-71)
Prior irradiation dose, median (IQR), cGy	6996 (6214-7020)
Proton reirradiation dose, median (IQR), CGE	66.0 (44.4-70.0)
Fractionated reirradiation dose, median (IQR), CGE	70 (66-70)
Quad shot reirradiation dose, median (IQR), CGE	44.4 (18.5-44.4)
No. of cycles, median (IQR)	3 (1-3)
Interval between irradiation courses, median (IQR), mo	22 (11-69)
Follow-up, median (IQR), mo	12.0 (5.8-26.0)
Follow-up of living patients, median (IQR), mo	24.5 (13.8-37.8)

Abbreviations: CGE, cobalt gray equivalent; KPS, Karnofsky performance status.

^a^ Scores range from 0 to 100, with higher scores meaning the patient is better able to carry out daily activities.

Among the early toxic effects assessed, 73 were grade 3 toxic effects and, most notably, 2 were grade 4 dysphagia and 4 were grade 4 dermatitis cases. Among the late toxic effects, 79 were potential grade 3 toxic effects in this cohort. Because many patients already presented with some level of toxic effects from prior treatment, the severity of late toxic effects observed is likely a combination of the 2 courses of radiation. Of these patients, 30 had dysphagia, 12 had osteoradionecrosis, 9 had fibrosis, 12 had trismus, 3 had hearing loss, 3 had fatigue, 3 had hoarseness, 2 had cranial neuropathy, 1 had dermatitis, 1 had temporal lobe necrosis, 1 had tinnitus, 1 had odynophagia, and 1 had grade 3 weight loss. There was also 1 potential grade 4 dysphagia as well as 3 potential grade 4 radiation dermatitis events. Five potentially treatment-related grade 5 bleeding events leading to death were also observed. An autopsy of 1 patient revealed innominate artery bleeding, which was believed to be related to the tracheostomy placement. Two cases had recurrent tumor at the site of the bleed; thus, it was unclear whether this recurrence was from treatment effect or the tumor. Another patient had a nonhealing neck wound, and careful review of records revealed a history of carotid pseudoaneurysm that may have also predisposed the patient to bleeding. One patient died 2 days after 4 cycles of quad shot with proton therapy. It is unlikely this was treatment related given the short interval from the end of radiation to the event; however, further details surrounding the death were unavailable (Table 2).

**Table 2.** Toxic Effects of Patients Receiving Proton Therapy Reirradiation

Toxic effect	No. of toxic effects			
	Grade 1	Grade 2	Grade 3	Grade 4^a^
**Acute grades**				
Mucositis	52	32	6	0
Xerostomia	90	22	1	0
Dysphagia	40	33	28	2
Odynophagia	25	3	2	0
Dysgeusia	21	4	0	0
Dermatitis	77	30	14	4
Pharyngitis	15	5	2	0
Nausea	8	3	0	0
Fatigue	84	26	4	0
Weight loss	24	21	4	0
Trismus	51	46	7	0
Fibrosis	50	23	5	0
**Late grades**				
Fatigue	60	20	3	0
Xerostomia	57	22	0	0
Dysgeusia	16	4	0	0
Dysphagia	25	26	30	1
Odynophagia	11	2	1	0
Otalgia	12	4	0	0
Tinnitus	12	1	1	0
Hearing loss	18	6	3	0
Lymphedema	22	6	0	0
Hoarseness	21	4	3	0
Cranial nerve palsy	5	1	2	0
Trismus	37	56	12	0
Fibrosis	57	33	9	0
Dermatitis	17	3	1	3
Nausea	4	0	0	0
Weight loss	17	11	1	0
Osteoradionecrosis	8	12	12	0
Temporal lobe necrosis	5	5	1	0
Hypothyroid	0	18	0	0
Carotid blowout syndrome	0	0	0	5

^a^ Potential grade 4 to 5 for late grades.

On Kaplan-Meier survival analysis, LC, LRC, OS, and PFS were all significantly better in the fractionated arm compared with those who received the quad shot regimen. Distant metastatic control, however, was not significantly different between the 2 groups. The 1-year OS was 28.5% (95% CI, 19.4%-38.3%) in the quad shot group and 66.6% (95% CI, 58.1%-73.8%) in the fractionated group. The 1-year LC was 61.6% (95% CI, 46.4%-73.6%) in the quad shot group and 71.8% (95% CI, 62.8%-79.0%) in the fractionated groups. At 2 years, the LC was 52.2% (95% CI, 34.6%-67.2%) in the quad shot group and 63.0% (95% CI, 53.3%-71.3%) in the fractionated group. The 1-year LRC was 58.5% (95% CI, 43.8%-70.6%) in the quad shot group and 62.0% (95% CI, 52.8%-70.0%) in the fractionated group (Figure).

**Figure.**
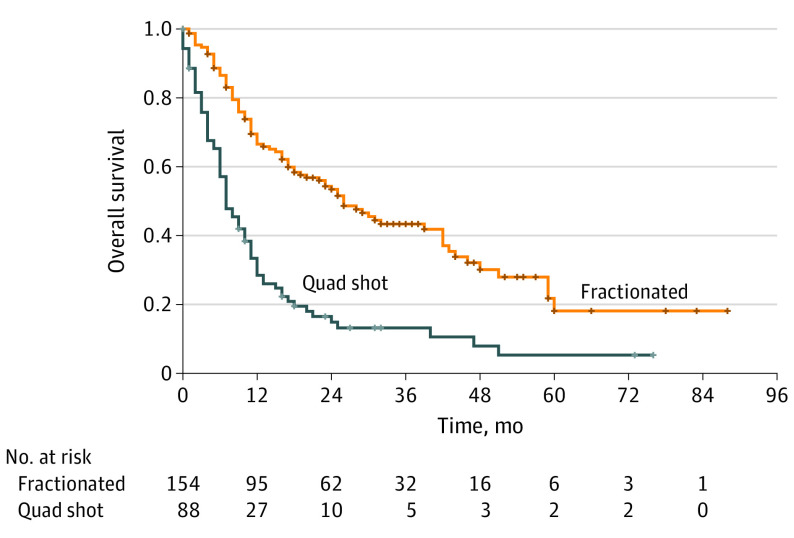
Comparative Overall Survival in Patients by Fractionation

On multivariate Cox proportional hazards regression modeling, higher PT-ReRT dose (hazard ratio [HR], 0.97; 95% CI, 0.95-1.00; *P* = .01) and receipt of salvage surgery (HR, 0.40; 95% CI, 0.22-0.74; *P* = .003) were significantly associated with improved LC (Table 3). On multivariate Cox proportional hazards regression modeling, a KPS score of 70 or higher (HR, 0.50; 95% CI, 0.25-0.99; *P* = .046) and receipt of salvage surgery (HR, 0.57; 95% CI, 0.39-0.84; *P* = .005) were associated with improved survival, whereas the quad shot compared with a fractionated regimen was associated with worse survival (HR, 1.97; 95% CI, 1.36-2.86; *P* < .001) (Table 4).

**Table 3.** Cox Proportional Hazards Model for Local Control of Head and Neck Squamous Cell Carcinoma

Variable	Univariate		Multivariate		
	HR (95% CI)	*P* value	HR (95% CI)	*P* value
Prior irradiation dose, cGy	1.00 (1.00-1.00)	.03	1.00 (1.00-1.00)	.12
Proton reirradiation dose, CGE	0.98 (0.96-0.99)	.004	0.97 (0.95-1.00)	.01
Interval between irradiation courses	1.00 (0.99-1.00)	.21	NA	NA
Smoking or tobacco use				
Never	1 [Reference]	NA	NA	NA
<10 pack-years	0.92 (0.42-2.02)	.82	NA	NA
≥10 pack-years	0.73 (0.44-1.21)	.22	NA	NA
Disease status at the time of irradiation				
Locoregional	1 [Reference]	NA	NA	NA
Distant metastases	1.59 (0.39-6.50)	.52	NA	NA
Salvage surgery				
No	1 [Reference]	NA	1 [Reference]	NA
Yes	0.42 (0.25-0.71)	.001	0.40 (0.22-0.74)	.003
Concurrent systemic treatment with reirradiation				
No	1 [Reference]	NA	NA	NA
Yes	0.79 (0.49-1.27)	.34	NA	NA
Reirradiation fractionation				
Fractionated	1 [Reference]	NA	1 [Reference]	NA
Quad shot	1.66 (1.01-2.73)	.046	0.56 (0.26-1.22)	.14

Abbreviations: CGE, cobalt gray equivalent; HR, hazard ratio; NA, not applicable.

**Table 4.** Cox Proportional Hazards Model for Overall Survival

Variable	Univariate		Multivariate	
	HR (95% CI)	*P* value	HR (95% CI)	*P* value
Age	1.00 (0.99-1.02)	.62	NA	NA
Sex				
Male	1 [Reference]	NA	NA	NA
Female	1.12 (0.79-1.60)	.52	NA	NA
KPS score^a^				
<70	1 [Reference]	NA	1 [Reference]	NA
≥70	0.48 (0.25-0.95)	.04	0.50 (0.25-0.99)	.046
Smoking or tobacco use				
Never	1 [Reference]	NA	NA	NA
<10 Pack-years	0.75 (0.41-1.35)	.34	NA	NA
≥10 Pack-years	1.10 (0.78-1.54)	.59	NA	NA
Disease status at the time of reirradiation				
Locoregional	1 [Reference]	NA	1 [Reference]	NA
Distant metastases	1.80 (0.79-4.08)	.16	0.78 (0.33-1.86)	.58
Both	1.92 (1.18-3.12)	.009	1.47 (0.89-2.42)	.14
Interval between irradiation courses	1.00 (1.00-1.00)	.29	NA	NA
Salvage surgery				
No	1 [Reference]	NA	1 [Reference]	NA
Yes	0.44 (0.32-0.62)	<.001	0.57 (0.39-0.84)	.005
Concurrent systemic treatment with reirradiation				
No	1 [Reference]	NA	1 [Reference]	NA
Yes	0.75 (0.55-1.02)	.06	0.79 (0.57-1.09)	.79
Reirradiation fractionation				
Fractionated	1 [Reference]	NA	1 [Reference]	NA
Quad shot	2.70 (1.97-3.70)	<.001	1.97 (1.36-2.86)	<.001

Abbreviations: HR, hazard ratio; KPS, Karnofsky performance status; NA, not applicable.

^a^ Scores range from 0 to 100, with higher scores meaning the patient is better able to carry out daily activities.

## Discussion

In this single-institution cohort study representing, to our knowledge, the largest cohort of PT-ReRT for 242 patients with HNSCC, LC exceeded the outcomes of previous studies^7,8^ in a cohort of patients with recurrent disease. The 1-year LC for the entire cohort was 68.4%, and for those receiving a fractionated course of reirradiation, the 1-year LC was 71.8% and the 1-year OS was 66.6%. Receipt of salvage surgery significantly improved survival on multivariable analysis. Although the patients had higher LC and OS rates, both early and late grade 3 or higher toxic effects were also observed.

Treatment of recurrent or second primary head and neck cancers is challenging, with median survival of 10 months for patients receiving combination chemotherapy with cetuximab per the phase 3 EXTREME (Erbitux in First-Line Treatment of Recurrent or Metastatic Head and Neck Cancer) trial published in 2008.^9^ With the advent of immunotherapy, the median overall survival of patients receiving nivolumab in the recurrent setting was 7.5 months,^10^ 11.5 months with pembrolizumab alone, and 13.0 months with pembrolizumab plus chemotherapy in the KEYNOTE-048 (A Study of Pembrolizumab [MK-3475] for First Line Treatment of Recurrent or Metastatic Squamous Cell Cancer of the Head and Neck) study.^11^ With significant improvement over the EXTREME regimen, immunotherapy became the preferred first-line systemic therapy option in the recurrent and/or metastatic setting^12^; however, there is still room for substantial gains in LRC.

Locoregional control is an important factor for quality of life and survival. Surgical resection, if possible, remains the first treatment of choice.^12^ There are limited prospective studies evaluating the benefit of local treatment for recurrent head and neck cancer. The GORTEC (Groupe d'Etude des Tumeurs de la Tête et du Cou and Groupe d'Oncologie Radiothérapie Tête Et Cou) study by Janot and colleagues^7^ randomized 65 patients to salvage surgery with or without adjuvant chemoradiation and found significantly improved LRC and DFS in the latter group. A benefit in OS was not observed, which may be due to high toxicity rates with grade 3 complications in 28% of cases and late grade 4 in 39%. At the time, patients were treated with 2-dimensional or some 3-dimensional conformal techniques, which may be a contributing factor. Since then, more advanced technology has allowed a dosimetric advantage, translating into improved toxicity profiles, which could further translate into improved OS.

A previous study^8^ reported the experience of 257 patients with varying histologic types of cancer using 3-dimensional conformal and intensity-modulated radiation therapy (IMRT). The majority of patients received IMRT (78%) and salvage surgery (67%). At a median follow-up of 32.6 months, the 2-year LRC and OS rates were 47% and 43%, respectively. Since the inception of the proton therapy program at our institution in 2013, the current analysis, which consists of only HNSCC, shows that there has been improvement in 2-year LRC of 52% and 2-year OS of 53% in the fractionated cohort. Furthermore, the initial analysis included non-HNSCC histologic types, which had better outcomes.

The current study also shows improvement in outcomes compared with a pooled multi-institutional analysis by the Multi-Institution Reirradiation (MIRI) Collaborative of 412 patients of squamous cell carcinoma and no distant metastases treated with IMRT or volumetric modulated arc therapy.^13^ The 2-year OS for the MIRI cohort was 40% compared with the 2-year OS of 53% in the current study. Although grade 3 toxicity numbers were high in the current study, our cohort had considerably higher OS, and the current study design had independent multidisciplinary physicians (radiation and head and neck surgery) to thoroughly verify and record toxic effects. In the MIRI study,^13^ recursive partitioning analysis showed intervals between radiation courses, salvage surgery, and organ dysfunction to be important factors associated with OS.

The study^14^ at the University of Texas MD Anderson Cancer Center of 60 patients from 2011 to 2015 who received PT-ReRT revealed no significant impact of salvage surgery or concurrent chemotherapy on outcomes. With a median follow-up of 13.6 months, 1-year LRC was 81%, 1-year OS was 81%, 1-year PFS was 60%, and 1-year DMC was 75%. Of note, although most patients (n = 40) had squamous cell carcinoma, 20 patients had non-SCC histologic types. Conversely, a multi-institutional study of 61 patients by McDonald et al^15^ found that surgery was a significant factor associated with improved OS. In this previous study,^15^ median OS was 16.5 months, 2-year OS was 33%, 2-year LC was 80% and 2-year DMC was 62%. A Russian study^16^ of 30 patients treated for unresectable disease from 2015 to 2020 with a median follow-up of 21 months reported a 1-year LC of 53%, 1-year PFS of 22%, and 1-year OS of 74%. Small cohort sizes are likely contributing to the varied range of outcomes observed with our survival outcomes comparable to the aforementioned studies (eTable in Supplement 1).

The toxic effects observed in the current study were not low, and because longer survival was observed compared with our IMRT experience, it is probable that patients are surviving long enough to develop late effects that would not have been seen previously. Similar to past reports^7,8,14^ of head and neck reirradiation, some deaths in our cohort may have been treatment related. Although proton therapy can limit the dose to the normal tissue compared with photon-based therapy due to its unique Bragg peak beam characteristics, it is still depositing high dose in the reirradiated field. For example, some patients had heavy bleeding, of whom at least 5 died from carotid blowout syndrome within the treatment field where patients had already received prior full-dose radiation. With the advent of proton therapy, we were more inclined to treat patients who were previously not candidates for reirradiation using photon-based reirradiation, including patients who were within 6 months of prior reirradiation. Unfortunately, there is little consensus on dose constraints to organs at risk in the reirradiation setting. Therefore, conservative approaches should be taken to minimize target volumes, using tools such as coregistered imaging with magnetic resonance imaging and positron emission tomography to optimize our understanding of where the highest risk of recurrence may be. These approaches may allow smaller treatment volumes, which in turn is likely to improve the toxicity profile. Incorporation of prior radiation plans, including fusion of Digital Imaging and Communications in Medicine radiotherapy files to generate a sum plan, is recommended if available in order to evaluate total cumulative doses and better optimize the reirradiation plan.

This cohort study is unique among reirradiation studies in that only patients with HNSCC were included, so histologic type could be controlled for. The decision needs to be made on the importance of LC versus toxic effects that may result from PT-ReRT. The Memorial Sloan Kettering Cancer Center is currently enrolling patients in a prospective study to evaluate outcomes and toxic effects. Lastly, the ongoing ECOG-ACRIN 3191 (Phase II Randomized Trial of Adjuvant Therapy with Pembrolizumab after Resection of Recurrent/Second Primary Head and Neck Squamous Cell Carcinoma with High Risk Features) will also elucidate the benefit of local therapy for recurrent head and neck cancer because it is randomizing patients who have undergone salvage surgery to 1 of 3 groups, including fractionated chemotherapy with reirradiation, reirradiation with immunotherapy, and immunotherapy alone.

### Limitations

As with any retrospective study, the limitations of this analysis are related to the inherent biases that are associated with a single-institution, nonrandomized cohort, including challenges with medical record review and the availability of data. Capturing an accurate assessment of toxic effects is particularly difficult in the reirradiation setting because many patients present with adverse effects from their first course of treatment; thus, it is difficult to ascertain when exactly events occurred and to quantify the effect from the second course of radiation. Furthermore, the toxic effects associated with the first course of radiation were not well documented, and many patients presented with preexisting trismus, fibrosis, dysphagia, fistulas, xerostomia, and hypothyroidism. Still, every effort was made to ascertain possible treatment effects by reviewing cases with the involved head and neck surgeon and medical oncologist so that there was consensus on toxic effects. Furthermore, toxic effect rates were not possible to establish due to data recording and difficulty with ascertaining when exactly the effects occurred. For example, rates of complications, such as temporal lobe necrosis, are not possible to establish without complete data on the number who received radiation to that site. Similarly, patients who had severe dysphagia after their first course of radiation may have required a gastrostomy and may still be dependent on it, so determination of the effect of a second course of radiation on dysphagia in those patients was not possible.

## Conclusions

This cohort study found that patients treated with proton therapy in a previously irradiated area of the head and neck may live longer compared with those receiving historical photon-based reirradiation. However, it is important to discuss the early and late complications that patients can still experience in the reirradiated tissue. Future studies are necessary to improve patient selection and derive optimal cumulative dose constraints for planning parameters to ultimately improve the therapeutic ratio.

## Notes

Supplement 1. **eTable.** Head and Neck Proton Re-irradiation Literature

Supplement 2.
Data Sharing Statement
