# Supplementary material for: Evaluation of Proton Therapy Reirradiation for Patients With Recurrent Head and Neck Squamous Cell Carcinoma
Source: JAMA Netw Open. 2023 Jan 23;6(1):e2250607. doi: 10.1001/jamanetworkopen.2022.50607 (PMC9871797; doi:10.1001/jamanetworkopen.2022.50607)
Supplement: Supplement 1. — eTable. Head and Neck Proton Re-irradiation Literature [file jamanetwopen-e2250607-s001.pdf]

Supplementary Online Content

Lee A, Woods R, Mahfouz A, et al. Evaluation of proton therapy reirradiation for patients with recurrent head and neck squamous cell carcinoma. *JAMA Netw Open*. 2023;6(1):e2250607. doi:10.1001/jamanetworkopen.2022.50607

eTable. Head and Neck Proton Re-irradiation Literature

This supplementary material has been provided by the authors to give readers additional information about their work.

© 2023 Lee A et al. *JAMA Network Open.*

**eTable.** Head and Neck Proton Re-irradiation Literature							
**Study**	**Institution**	**Year**	**N**	**Median Proton ReRT Dose (CGE)**	**OS**	**LC**	**Late G3+ Toxicity (%)**
McDonald et al. (2016)[15]	Indiana University (USA)	2004-2014	61	66	Median 16.5 mo<br>2-yr 32.7%	2-yr 80.3%	24.6%<br>3 treatment-related deaths
Hayashi et al. (2017)[17]	Southern Tohoku Proton Therapy Center (Japan)	2009-2014	34	50	1-yr 62%<br>2-yr 42%	1-yr 77%<br>2-yr 60%	G3 ORN (3%)<br>No G4 or G5 toxicities
Phan et al. (2016)[14]	University of Texas MD Anderson (USA)	2011-2015	60	66 (definitive)<br>61.5 (adjuvant)	1-yr 81.3%<br>2-yr 69.0%	1-yr 68.4%<br>2-yr 55.9%	20.0%<br>2 potential treatment-related deaths
Dionisi et al. (2019)[18]	Proton Therapy Unit, APSS (Italy)	2015-2018	17	60	1.5-yr 54%	1.5-yr 67%	23.5%<br>6% potential fatal bleeding
Gordon et al. (2021)[16]	A. Tsyb Medical Radiological Research Center (Russia)	2015-2020	30	57.6	Median 16 mo<br>1-yr 73.4%<br>2-yr 8.4%	1-yr 52.6%<br>2-yr 21.0%	16.6%<br>1 treatment-related death

© 2023 Lee A et al. *JAMA Network Open.*
