# Supplementary material for: Evaluation of Proton Therapy Reirradiation for Patients With Recurrent Head and Neck Squamous Cell Carcinoma
Source: JAMA Netw Open. 2023 Jan 23;6(1):e2250607. doi: 10.1001/jamanetworkopen.2022.50607 (PMC9871797; doi:10.1001/jamanetworkopen.2022.50607)
Supplement: Supplement 2. — Data Sharing Statement [file jamanetwopen-e2250607-s002.pdf]

Data Sharing Statement

Lee. Evaluation of Proton Therapy Reirradiation for Patients With Recurrent Head and Neck Squamous Cell Carcinoma. *JAMA Netw Open*. Published January 23, 2023. doi:10.1001/jamanetworkopen.2022.50607

Data
Data available: No
